# Supplementary material for: Development of a Nomogram Predicting the Risk of Persistence/Recurrence of Cervical Dysplasia
Source: Vaccines (Basel). 2022 Apr 9;10(4):579. doi: 10.3390/vaccines10040579 (PMC9029732; doi:10.3390/vaccines10040579)
Supplement: Supplementary file 1 [file vaccines-10-00579-s001.zip › vaccines-1656238-SI update.pdf]

Figure S1: Follow-up after primary conization

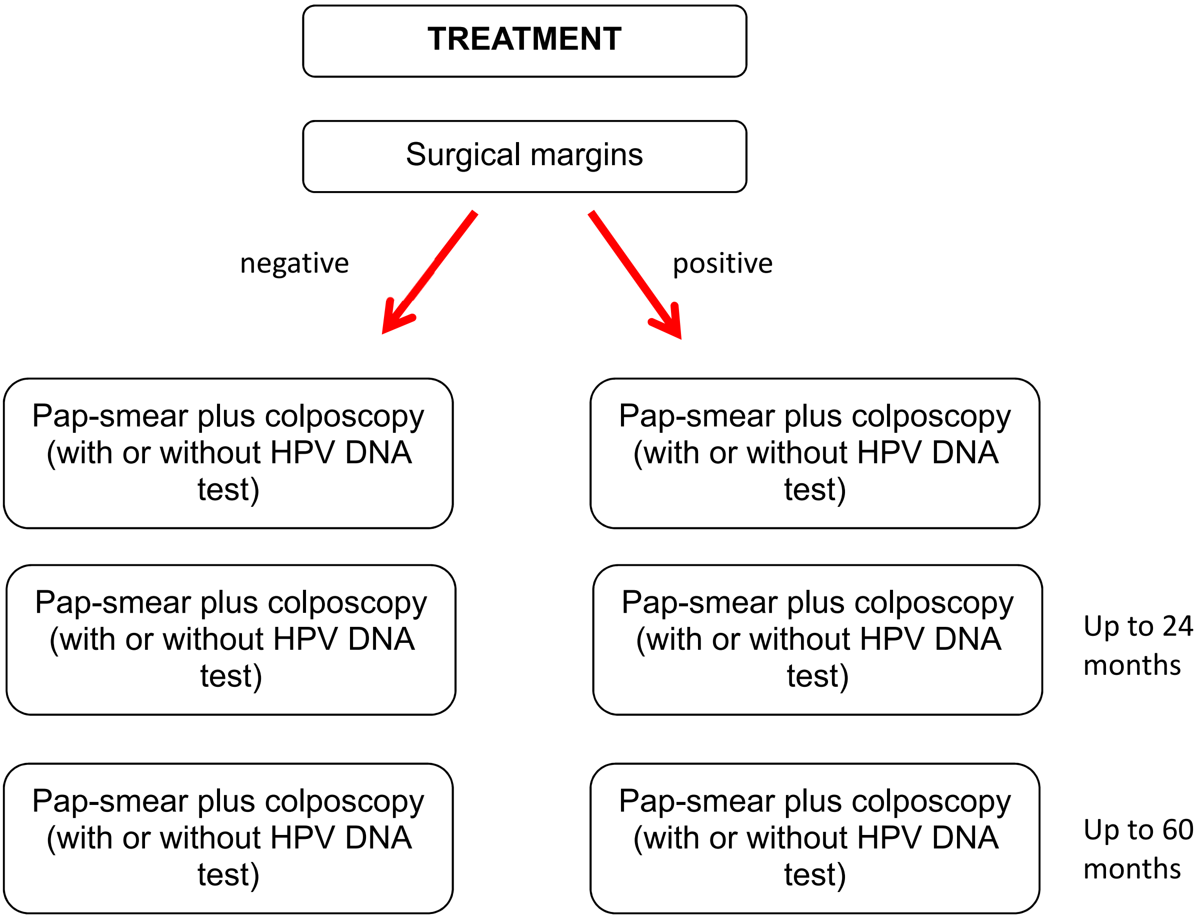

In case of positive results patients had cervical biopsy or endocervical curettage

Figure S2: Risk of persistence/recurrence according to vaccination status

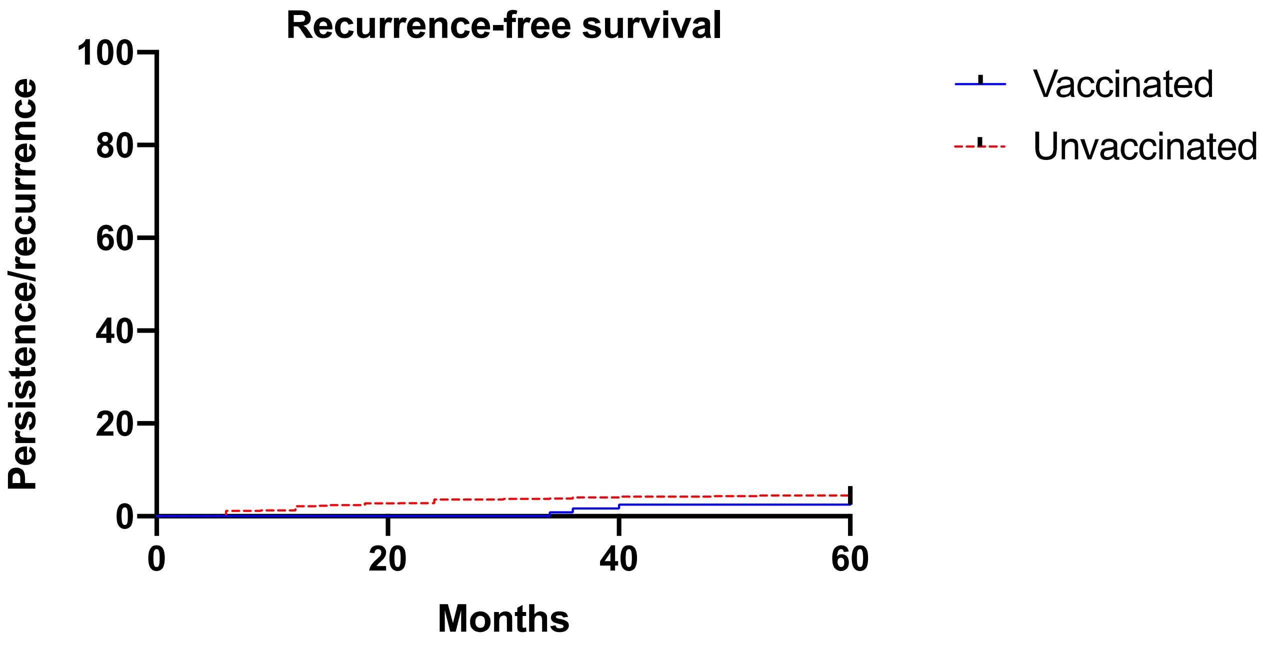

5-year risk of developing cervical dysplasia persistence/recurrence according to the HPV vaccination status (p=0.302)

Figure S3: Risk of persistence/recurrence according to patients' age

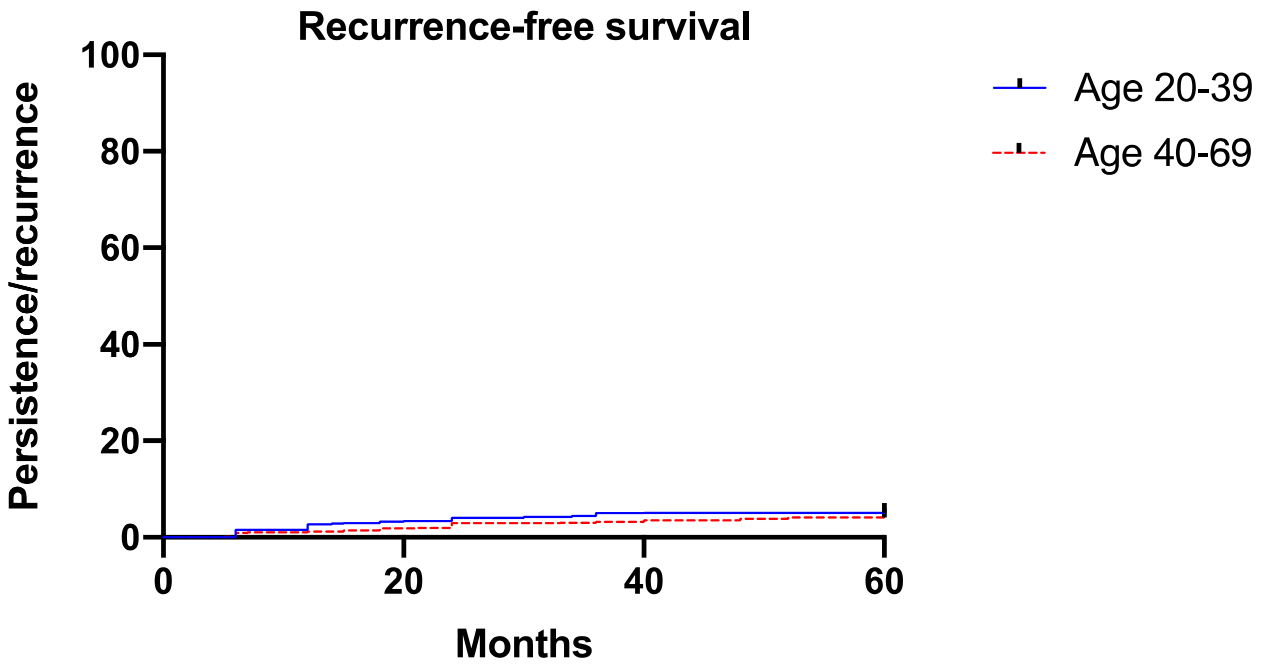

5-year risk of developing cervical dysplasia persistence/recurrence according patients' age (p=0.218)

Figure S4: Diagnostic performance of the model

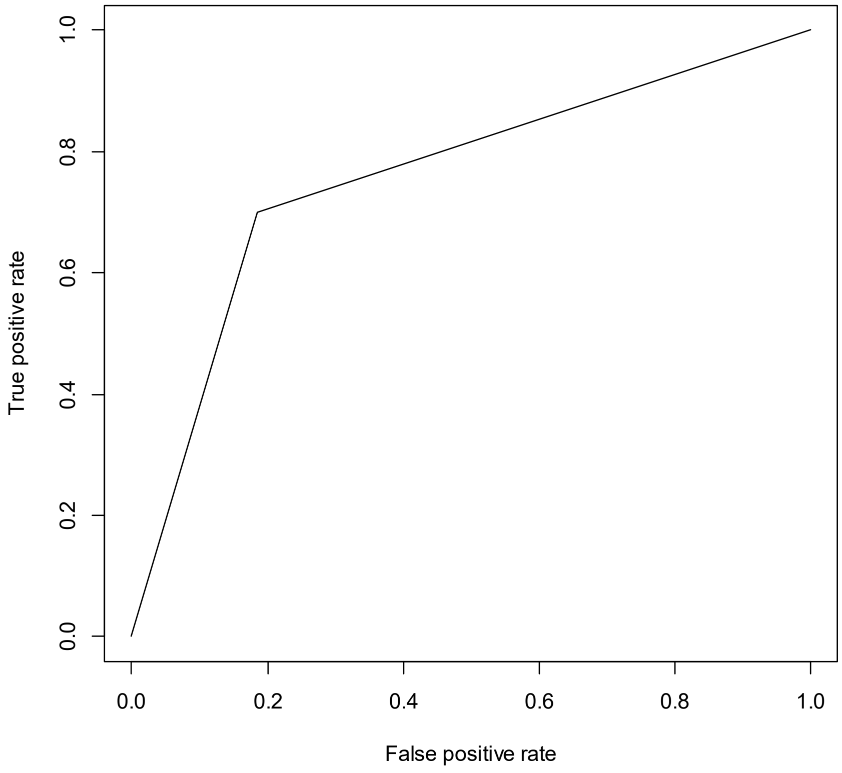

AUC= 0.7576433

Figure S5 : Predictive value of the model

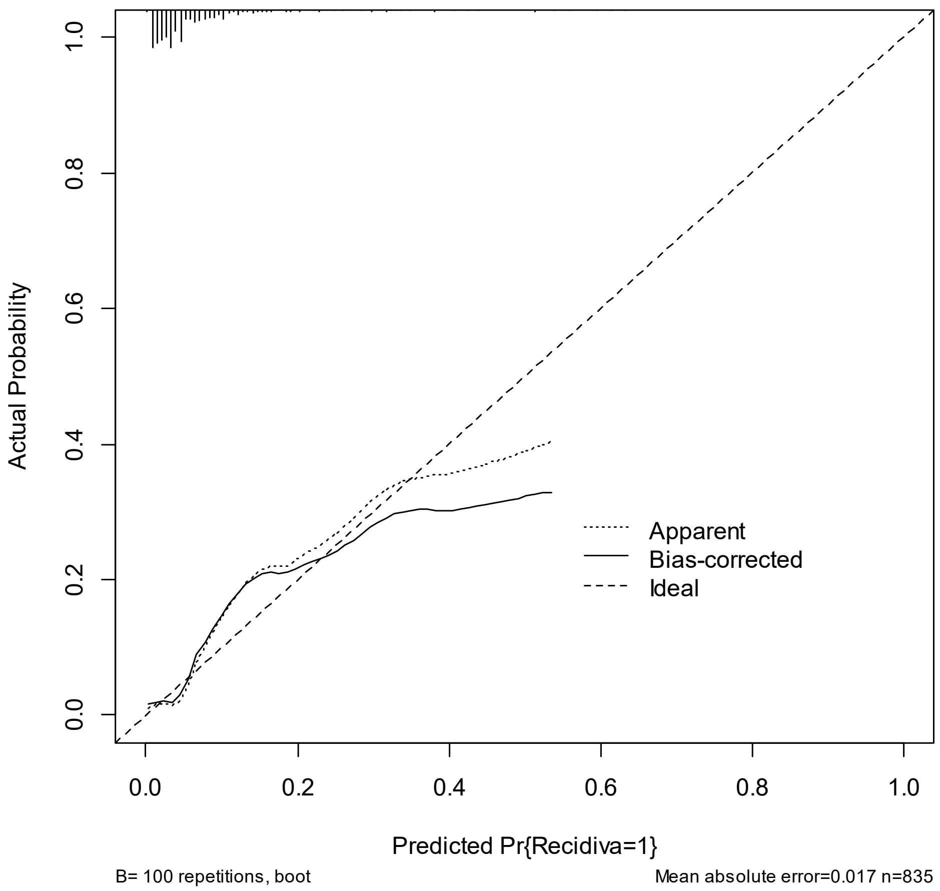


```
Mean absolute error=0.017   Mean squared error=0.00069
0.9 Quantile of absolute error=0.042
```

Table S1: Logistic model

**Model Likelihood**		**Discrimination**		Rank
	**Ratio Test**		Indexes	
LR chi2	56.08	R2	0.190	C
d.f.	9	G	1.283	Dxy
Pr(> chi2)	<0.0001	Gr	3.607	Gamma
		Gp	0.058	Tau-a
		Brier	0.046	

Table S2: Risk assessment according to various variables.

Variables	Value	Points
Age	10	100
	20	88
	30	75
	40	63
	50	50
	60	38
	70	25
	80	13
	90	0
Reason for conization	CIN2	0
	CIN3	29
HPV type involved	16/18	56
	Other high-risk	63
	No high-risk	0
Surgical procedure	LEEP	10
	Laser conization	0
Ectocervical margins	Negative	0
	Positive	14
Endocervical margins	Negative	0
	Positive	44
HPV persistence	No	0
	Yes	25
Adjuvant vaccination	No	65
	Yes	0

Table S3: Risk of recurrence according to the nomogram

Total Points	Risk of recurrence
144	0.005
209	0.050
230	0.100
252	0.200
267	0.300
279	0.400
290	0.500
301	0.600
314	0.700

Table S4: Baseline characteristics of the validation cohort

	Study population (n=549)
Age, years	46 (19, 71)
BMI	23 (17, 41.0)
Menopause	
No	355 (64.7%)
Yes	194 (35.3%)
Reason for conization	
CIN2	139 (25.3%)
CIN3	410 (74.7%)
HR HPV involved	
No	49 (8.9%)
Yes	500 (91.7%)
Positive margins	
Endocervical	33 (6.0%)
Ectocervical	23 (4.2%)
Vaccination after conization	
No	498 (90.7%)
Yes	51 (9.3%)
HPV persistence	
No	401 (73.1%)
Yes	148 (26.9%)

Data are reported as median (range) and number (%); Abbreviation: BMI, body mass index; CIN, cervical intraepithelial neoplasia; HPV, human papillomavirus
